# Performance Evaluation Method of Public Administration Department Based on Improved DEA Algorithm

**DOI:** 10.1155/2022/2338680

**Published:** 2022-09-30

**Authors:** Qing Guan, Shuilong Zou, Hong Liu, Qing Chen

**Affiliations:** ^1^School of Economics and Management, Nanchang Normal College of Applied Technology, Nanchang, Jiangxi 330108, China; ^2^College of Electronic and Information Engineering, Nanchang Normal College of Applied Technology, Nanchang, Jiangxi 330108, China; ^3^Department of Information Engineering, Gongqing College of Nanchang University, Gongqingcheng, Jiangxi 332020, China; ^4^Business College, Nanchang Jiaotong Institute, Nanchang, Jiangxi 330100, China

## Abstract

This paper proposes a performance evaluation method of public administration departments based on the improved DEA algorithm, which solves the quality problem of performance evaluation of public administration departments and lays a foundation for the long-term development and performance improvement of public administration departments. The DEA model is the core to sort out the performance evaluation methods of public administration departments. From the perspective of DEA algorithm, whether DEA is effective is an important content that public administration departments must consider when carrying out performance administration, and public service satisfaction is the core parameter obtained by DEA algorithm. That is to say, by optimizing public services and improving service satisfaction, the public administration department can further improve the quality of performance evaluation. Therefore, we can carry out performance evaluation of public administration departments from the satisfaction of public services. The empirical research conclusion shows that according to the effective judgment theorem of DEA, it can be concluded that among the eight social security departments, there are five departments that can achieve DEA effectiveness, namely department 2, department 3, department 4, department 5, and department 6. There are three non-DEA valid ones, namely, department 1, department 7 and department 8. The public satisfaction and the total service cost will affect the performance quality to a certain extent, and only by balancing various influencing factors can the performance evaluation quality of the public administration department be maximized.

## 1. Introduction

Nowadays, with the continuous development of social economy, as an important part of supporting China's social stability, public administration departments need to improve the quality of work from the level of performance. First of all, with the establishment and continuous improvement of the market economic system, the reform of the public sector management system and methods has been mentioned in a more prominent position. Performance evaluation is one of the themes of contemporary public management development, and public sector performance evaluation has also become the difficulty and core of the management model reform. DEA algorithm is the key to the performance evaluation of public administration departments, and combined with DEA algorithm, the accuracy of performance evaluation can be maximized and the future development direction of performance optimization of public administration departments can be understood. The DEA algorithm is mainly used in multi-input/multioutput system models and has outstanding advantages for evaluating the relative effectiveness of the same type of departments, which is its great advantage. For example, textile factories of the same type, colleges and universities, hospital libraries, and functional departments with the same nature of government work as if each member is a decision-making unit. Therefore, it is necessary to analyze the performance evaluation methods of public administration departments with DEA algorithm as the core, so as to make the performance evaluation of these departments better.

**Figure 1:**
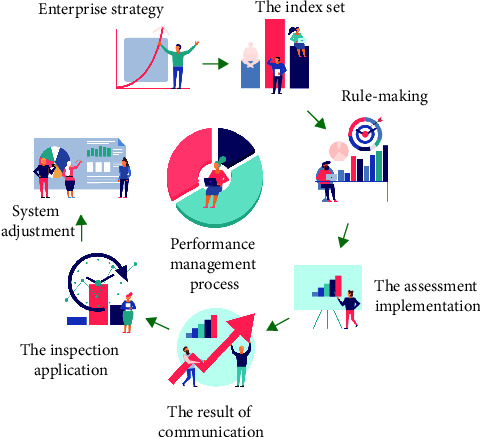
The trajectory of public administration activities.

Zhao et al. believed that the difference between management performance and performance management was significant in public administration. Management performance was the result of organizational efficiency and benefit generated by human beings in the process of social activities through management means. The level of management performance depended on the scientific level of management activities [1]. According to Zhuravel performance management was a kind of taking the ultimate benefit as the value standard of organizational management activities. Based on the establishment of organizational performance objectives, the scientific management method of optimal management efficiency and benefit was realized through the scientific matching combination of cost, input, output, efficiency, and benefit [2]. Kim et al. believed that performance management differed from traditional management methods in that it emphasized the final effect of management activities and the objective control and evaluation correction mechanism of management process, and advocated effect control rather than procedural control [3]. For the track of public administration activities, Yesennikov believed that the public administration department referred to the public organization using public power to manage social public affairs in accordance with the law [4]. Here, the public department studied in the research referred to the party and government institutions with the function of public affairs management. Vasileios believed that the essential attribute of the public department determined the characteristics of the management activities of the public department, which was usually manifested as the management of the authoritative administrative mode [5]. Therefore, Ozdogan et al. believed that whether the management activities of the public department were fair and just, whether they met the interest requirements of citizens, and whether the management and service of the public department achieved good performance should and must be checked and supervised by the society [6]. Moradi and Barakat believed that the definition of performance evaluation of the public department was very broad, and the mainstream view held that performance evaluation of public administration department referred to the activity of measuring and evaluating government performance within a certain period of time in order to improve the performance of government behavior and enhance control [7]. Tureta et al. believed that government performance evaluation could be regarded as the evaluation and grading of the performance reflected by the input, output, midterm results and final results in the process of government public department management based on the analysis and judgment of efficiency, capacity, service quality, public responsibility, and public satisfaction [8]. Dwivedi et al. believed that according to the implementation method, role, and purpose of performance evaluation, in the research, it was believed that performance evaluation of public department was to compare the actual work results of public department with performance objectives [9]. Wang et al. believed that the results of the actual work of the organization should be evaluated based on analysis and judgment from the aspects of work efficiency, management ability, management cost, and public satisfaction, so as to make a comprehensive evaluation of the performance of the organization [10]. The trajectory of public administration activities is shown in Figure 1.

## 2. Methods

**Figure 2:**
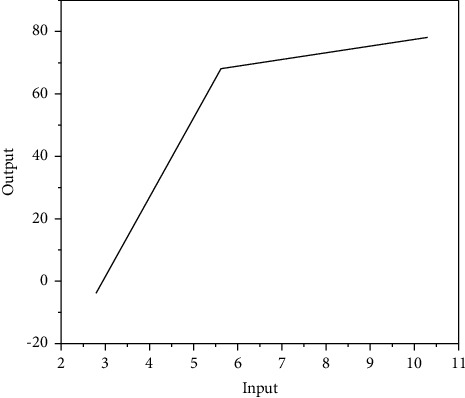
Basic forms of data envelopment analysis.

Data envelopment analysis (DEA) method uses the linear programming technology of operations research to analyze the envelope surface of input data and output data of different decision-making units. By judging the relationship between the observed values of different decision-making units and the position of the efficiency frontier, the efficiency problem is investigated. It is a quantitative analysis method to evaluate the relative effectiveness of comparable units of the same type based on multiple input indicators and multiple output indicators using linear programming. Up to 1996, there have been more than 600 related researches on the empirical application of DEA method, and DEA method has shown incomparable advantages for both for-profit and nonprofit organizations [11]. A university used DEA method to evaluate the operating performance of 24 airports in the United States. The four input variables selected were airport operating cost, number of employed staff, number of boarding gates, and number of runways. The selected five output variables were operating income, passenger flow, commercial flight flow, general flight flow, and total cargo transport. The empirical research results showed that 22 airports achieved both technical efficiency and scale efficiency in five years of operation, accounting for 92% of the total number of decision-making units. The research showed that the operating efficiency of American airports was generally good. The basic form of data envelopment analysis is shown in Figure 2 [12, 13].

DEA method was used to evaluate the efficiency of different decision-making units (DMU) with multiple inputs and outputs [14]. After the basic idea of DEA method was put forward in 1957, it was expanded to multi-input and multioutput efficiency evaluation model (CR model) under fixed scale returns in 1978. In 1984, the DEA model was improved and became an evaluation model covering technical efficiency and scale efficiency under variable returns to scale (CB model) [15]. In 1985, the academic circle proposed the sensitivity analysis of DEA mathematical model to recalculate efficiency values by gradually reducing input and output variables or the number of decision-making units, so as to conduct sensitivity analysis. DEA method used linear programming technology to project all the inputs and outputs of DMU into the efficiency space and work out the efficiency frontier. This efficiency frontier was the track connected by the most efficient production points of DMU. DEA is a linear programming model expressed as the ratio of output to input. It attempts to maximize the efficiency of a service unit by comparing the efficiency of a particular unit with the performance of a group of similar units providing the same service. In this process, some units that achieve 100% efficiency are referred to as relatively efficient units, while additional units with an efficiency score below 100% are referred to as inefficient units. The production points located on the track were the most efficient input–output combination and their efficiency value was 1. With full technical efficiency, namely, with the least input or the maximum output under the given input, the efficiency value and projection value of different DMU were calculated by using the relationship between the actual observed value of DMU and the position of the efficiency frontier [16]. The main data model of DEA method is as follows.

Parameter setting: Suppose there are *n* DMUs, each using *m* inputs *xi*(*i*=1, 2......, *m*) to produce *s* outputs *yr*(*r*=1, 2......, *s*). *θ*_*k*_ represents the potential amount of all DMU_k_ inputs that can be reduced in equal proportion. Weight *λ*=(*λ*_1_, *λ*_2_, ⋯*λ*_*n*_) represents a polyhedron vector that joins all data.

The fractional programming is shown in

(1) $$\begin{array}{c} \max h_{k}=\frac{\sum_{r=1}^{x}uy}{\sum_{i=1}^{m}vx}\leq 1\text{.} \end{array}$$

The linear programming is shown in

(2) $$\begin{array}{c} \mathrm{Max}h_{k}=\overset{x}{\underset{r=1}{\sum }}U_{r}y_{rk}\text{.} \end{array}$$

The dual programming is shown in

(3) $$\begin{array}{c} \overset{n}{\underset{j=1}{\sum }}y_{j}\lambda_{j}-s^{i}=y_{k}\text{.} \end{array}$$

Formulas (1) to (3) are the basic development and evolution processes of DEA mathematical model. The dual programming formula is CR model in DEA method. If the optimal solution of CR model $\overset{o}{\theta }$ < 1, it is believed that the $\overset{o}{j}$th decision unit DEA is invalid. If the optimal solution of the *C*^2^*R* model $\overset{o}{\theta }$ = 1, the $\overset{o}{j}$th decision unit is weak DEA efficient. If the optimal solution of CR model $\overset{o}{\theta }$ = 1, *s* = 0, *s*′ = 0, then the$\overset{n}{j}$th decision unit is DEA effective. To judge the effectiveness of DMIU operations is essentially to judge whether it is on the efficiency frontier.

Before the reform and opening up, performance evaluation of public administration departments in China mainly focused on individual performance evaluation. After the reform and opening up, China's public department has undergone five major adjustments, with great changes in its management mode, organizational structure and operation, and management mechanism. With the deepening of the institutional reform, the content of reform should be refined along with scientific progress. Especially after China's entry into the World Trade Organization, it objectively urges China's public management system to speed up the integration with international practices. Through the previous reforms, on the premise of basically establishing the public management system of socialist market economy, absorbing, introducing, and learning advanced management concepts, mechanisms, and methods of developed countries will become an important part of public reform [17].

At present, the research topics of public department performance evaluation mainly focus on 10 aspects (as shown in Table 1). It mainly covers the performance evaluation of local public departments in China, evaluation and financial revenue and expenditure, evaluation subjects, evaluation system reform, evaluation value orientation, evaluation empirical research, evaluation mode innovation, evaluation mechanism innovation, and introduction of evaluation methods of performance evaluation of public departments in various countries. The types of literature on performance evaluation of public administration departments are shown in Table 1.

Efficiency is a term used to describe the ability of a system to convert inputs into outputs. It is the ratio of the actual capacity of the system to the optimal level of capacity that should be achieved.

The efficiency calculation formula is shown in

(4) $$\begin{array}{c} S=\frac{R}{T}\text{,} \end{array}$$

*S* is efficiency. *R* is actual capability. T is the best ability.

The efficiency is usually no more than 1. When the concept of efficiency is used in the public management department, efficiency represents the operational capacity of the department; therefore, it is also called operational efficiency. Operational efficiency refers to how effectively an enterprise uses its assets. The more output per unit time, the higher the efficiency and the stronger the enterprise's profitability. It reflects the capital turnover of the enterprise. The level of operational efficiency depends on the quality of business operations and the level of management. Microeconomics defines “the maximum output an economy can get under the condition of established technical knowledge and input quantity” as production-possibility frontier (PPF). Efficiency is explained as “when an economy cannot obtain more output without reducing one kind of output, that is, when it is on the production possibility boundary, it is called that the production of the economy is efficient” [18]. The level of efficiency depends on a variety of factors, such as the size of the management department, the combination of input factors (resources), system, technical conditions, market competition, psychological factors of enterprise personnel (enterprise culture), and so on. That is to say, changes in these factors will cause changes in the operational efficiency of the department. There are many indicators describing the efficiency. The following mainly introduces technical efficiency (TE) related to the system, organization or technology, allocative efficiency (AE) related to the combination of inputs, scale efficiency (SE) related to department scale, and the overall efficiency (OE) and pure technical efficiency (PTE) related to the above three kinds of efficiency. The first three efficiencies are described under the condition of constant return to scale (CRS).

**Figure 3:**
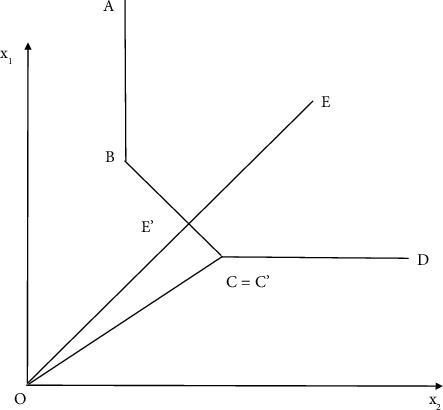
Efficiency value of DMU.

To account for OE, TE, and AE, we assume that a management uses two factors of production, *X*1 and *X*2, to carry out management activity *Y*, assuming constant scale. The curve UU′ represents the isoquant curve of the management department under the condition of constant returns to scale and existing technology as shown in Figure3. Obviously, it is impossible for the input combination at the lower left of the curve UU′ to be used to produce the same output *Y*, and it is inefficient for the input combination at the upper right of the curve UU′ to be used to produce the output *Y*. The straight line PP is an isocost line of the management department, it is tangent to UU, and the tangent point is *C*. The slope of the straight line PP′ represents the value ratio of the two input factors.

The technical efficiency (TE) of management department at point *A* is defined as the ratio of optimal investment (OB) to actual investment (OA) under the assumption that return to scale remains unchanged, that is, TE = OB/OA. It reflects the gap between the input combination at point A and the minimum input combination represented by UU at the equal output line under the condition of equal output. If TE = 1, it indicates that management technology is effective (Technical efficiency means that management cannot increase any output unless it increases an input or decreases an output. That is, the output cannot be increased by recombination of input or output with existing resources.), namely *A* point on the isoquant curve. If TE < 1, it indicates that technology *A* is invalid.

The allocation efficiency (AE) of the management department at point *A* can be defined as follows: AE −0D/OB, where *D* is located on the isocost line PP. This is because compared with point *C* where both technical efficiency and allocation efficiency are effective, the management department producing at point *B* can improve the allocation efficiency by changing the combination of input elements. BD obviously, the management department produces on the minimum cost line (such as point *C*)), its configuration efficiency is 1. Configuration effective refers to when the element is at a given price, the cost of the investment portfolio achieves the minimum. Otherwise, the configuration is invalid. It is not only an integral part of the public sector's own performance management system but also an important means for citizens' representative institutions and the society to supervise and control the public sector. That is to say, public sector management performance evaluation is not only a management and monitoring tool but also a process of using the tool to carry out verification and monitoring activities.

**Figure 4:**
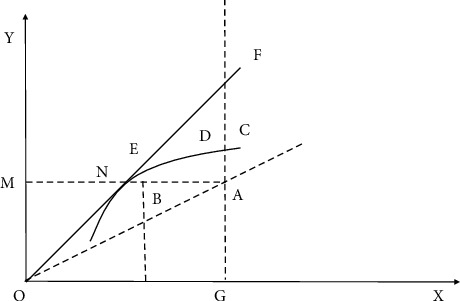
Pure technical efficiency and scale efficiency.

The overall efficiency (OE) of the management department is equal to the product of technical efficiency and configuration efficiency: OE − TE × AE = (OB/OA) × (OD/OB)*m* OD/OA. The overall efficiency reflects the ratio of the ideal minimum cost of management to the actual cost of producing the current level of output. When the management department is at point *C*, its overall efficiency is 1, that is overall effective. Otherwise, it is overall ineffective. Pure technical efficiency and scale efficiency are shown in Figure 4.

In Figure 4, we relax the assumption of constant scale and illustrate the concepts of pure technical efficiency and scale efficiency, and their relationship to technical efficiency, using the single-input and single-output cases. In Figure 4, NEF represents the efficient production frontier with constant returns to scale, and BEDC represents the efficient production frontier with variable returns to scale. The pure technical efficiency (PTE) of a managerial sector is the distance between the managerial sector under consideration and the frontier of efficient production when the returns to scale are variable: PTE = MB/MA. The scale efficiency (SE) of the management sector is the distance between the efficient production frontier of constant scale and the efficient production frontier of variable scale.

The scale efficiency formula is shown in

(5) $$\begin{array}{c} SE=\frac{MN}{MB}\text{.} \end{array}$$

Management departments that achieve pure technical efficiency may not make full use of scale return technology, so it is possible to improve efficiency. The management department with constant return to scale has scale efficiency. The reason is simple. If the management department is in the stage of increasing return to scale, it can increase the output–input ratio of the management department by increasing its scale, and vice versa. Therefore, the management department can have scale efficiency only when the return to scale remains unchanged. It can be found that technical efficiency (TE) can be divided into scale efficiency (SE) and pure technical efficiency (PTE).

The technical efficiency formula is shown in

(6) $$\begin{array}{c} TE=SE*PTE\text{.} \end{array}$$

DEA model is the core content of DEA method. The earliest DEA model is the *C*^2^*R* model, which is an ideal and effective tool to study the “technical effectiveness” of multiple DMU. The initial DEA model *C*^2^*R* is a fractional programming, which can be transformed into an equivalent linear programming problem by using *C*2 transformation. This fractional programming extends the definition of scientific and engineering efficiency to the concept of relative efficiency of multi-input and multioutput systems. By analyzing the duality theory of linear programming, a dual programming can be obtained, which has its economic meaning and is related to the production possibility set and the corresponding production frontier. Assume that there are *n* departments or decision-making units (DUS), which are all comparable. Each DMU has m types of inputs (representing its consumption of “resources,” similar to factors of production in microeconomics) and *s* types of outputs (which are indicators of the “effectiveness” of the DMU after it has consumed resources). The efficiency value of DMU is shown in Figure 3.

At this point, decision-making unit *j*(*j*=1,2, ⋯⋯, *n*). The input vector of *n* is denoted as *X*_*j*_ = (*X*_1*j*_, *X*_1*j*_,…, *X*_*mj*_)^*y*^. The output vector is denoted as *Y*_*j*_ = *(Y*_1*j*_, *Y*_1*j*_,…, *Y*_*rj*_)^*y*^, where *X*_*ij*_ represents the total input of the *j*th decision making unit to the input of the *i*th type, *X* > 0. *Y*_*rj*_ represents the total input of the *j*th decision-making unit to the total output input of the RTH type output, *Y*_*rj*_>0.

The *C*^2^*R* model is shown in

(7) $$\begin{array}{c} C^{2}R\left\{\begin{array}{c} \max\frac{\underset{t}{U}\overset{k}{Y}}{\underset{t}{V}\overset{k}{X}}=\overset{I}{\underset{P}{V}},\\ \frac{\underset{t}{U}\overset{k}{Y}}{\underset{t}{V}\overset{k}{X}}\leq 1,\\ j=1,2,\cdots \cdots ,n,\\ u\geq 0,v\geq 0\text{.} \end{array}\right. \end{array}$$

The original *C*^2^*R* model is a fractional programming, which can be transformed into an equivalent linear programming form using the *C*^2^ transformation. By *V*^*t*^*X*_*k*_>0, *t* > 0 is obtained.

## 3. Experiment and Analysis

Managers are always under pressure to improve business performance, so they are constantly looking for efficient production and operation modes. DEA method provides managers with the direction and degree of improvement when transforming invalid units into effective units. According to the DEA algorithm, performance evaluation indicators should include input indicators and output indicators. The input index of the performance evaluation of this functional department refers to the occupation or use cost of the department's human, financial, material, and other resources, including the number of employees, the professional education level of the employees, the number of vehicles that can be deployed, the jurisdiction, the funds used, the geographical location, etc. In this chapter, the social security department of *X* City was selected for the empirical analysis. DEA method was used to evaluate the performance of the social security department. Corresponding countermeasures and suggestions based on the evaluation results were put forward [19].

**Figure 5:**
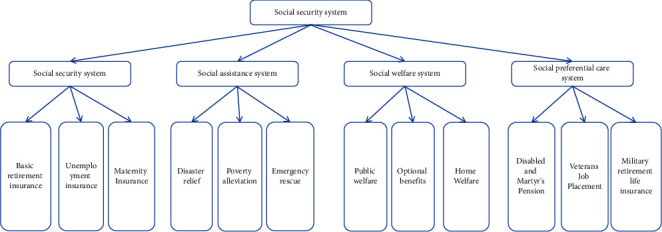
Social security framework.

Due to the loose management of social security departments, eight subdepartments of social security departments in different urban areas of *X* City were selected as research objects to conduct empirical research on their business performance. These eight departments were all located in *X* City. Because of the universality of social security, it was believed that the operating performance of social security departments had nothing to do with the urban areas, but had something to do with the urban population. Social security performance evaluation is based on the judgment of management efficiency, service quality, public responsibility, public satisfaction, etc., to evaluate the performance reflected in the input, output, and final results of social security institutions in the management process. In short, it is to evaluate the effect of social security responsibility implementation by setting up a series of indicator systems. In order to improve the convenience of analysis, eight departments were defined as department 1 to department 8, respectively. The social security framework is shown in Figure 5 [20].

There are three input indicators selected in this paper: *x*: the number of services and products (pieces); *X*_*q*_: the unit cost of each service (yuan/piece); *X*_*j*_: the number of complained services (pieces). There are also two output indicators: *Y*: departmental efficiency; *Y*_*q*_: mass service satisfaction. The unit cost of each service *X*2: unit service cost = total service cost/the number of services and products, the total service cost and the number of complained services.

Public service satisfaction *Y*, this data are obtained through field visits, surveys, and statistics. A total of 800 questionnaires were distributed in 8 departments, and 780 questionnaires were collected from randomly selected users during working hours. After statistics, 775 valid questionnaires were collected, with an average of 96 valid questionnaires for each department, and 88 valid questionnaires for the department with the least questionnaires. Statistical table of data of social security departments is shown in Table 2.

**Figure 6:**
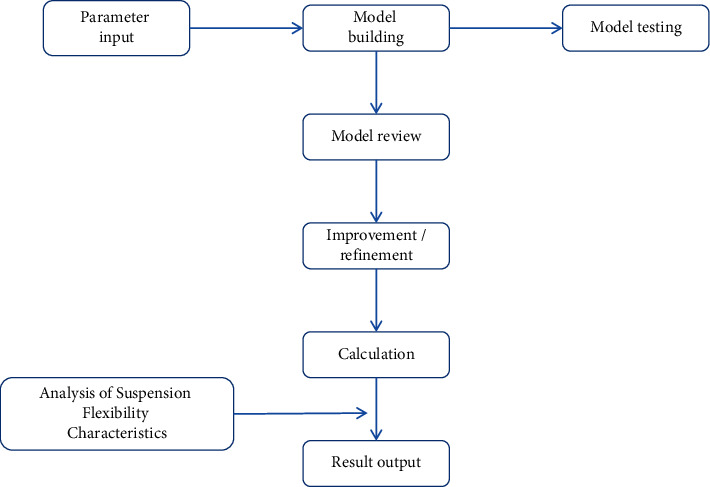
DEA model analysis process.

DEA method is to compare the relative efficiency between decision-making units by using mathematical programming model, while EXCEL software has the function of solving linear programming problems. So in the research, EXCEL software was used to solve DEA model. Input and output variables were confirmed according to different characteristics of different research objects and then they were input into EXCEL software system, which could make use of continuous cross calculation of variables of various types to form effective production front surface; and then the distance from each unit to the front surface was calculated to give the comprehensive score of each decision-making unit [21]. EXCEL software could make comparative analysis of any two decision-making units and could make the comparison difference visual effect. It could determine the performance of each unit of input and output elements and the percentage of difference with the front. It could confirm the best performance unit and the star shop. In order to use the DEA model to analyze business efficiency more accurately and scientifically, it is essential to carry out correlation analysis on indicators. To analyze the correlation between indicators, the principle of selecting indicators is the following: the input indicators and the output indicators should be as uncorrelated as possible. The input indicators and output indicators should be as relevant as possible. DEA model analysis process is shown in Figure 6.

In the three-input indicators of total service cost, unit cost of each service, and the number of services being complained about, and the two output indicators of departmental efficiency and service satisfaction, through correlation analysis, it can be found that the correlation coefficient between the input indicators can basically be maintained below 0.5, and the correlation coefficient between output indicators is also below 0.5. It showed that the correlation between input indicators and output indicators was relatively weak. If the correlation coefficient between the input index and the output index is above 0.5, it means that the correlation between the input index and the output index is relatively large. This conforms to the principle of index selection. Therefore, all six indicators are determined, and the correlation degree is relatively large. This conforms to the principle of index selection and divides the overall efficiency value of the department and the dummy variable value of each constraint condition. Taking department 1 as an example, the specific calculation formula is shown in

(8) $$\begin{array}{c} \left\{\begin{array}{c} \min\;\theta ,\\ \lambda_{1}Y_{11}+\lambda_{2}Y_{12}+\cdots +\lambda_{8}Y_{18}+S_{1}^{-}=\theta X_{11},\\ \lambda_{1}Y_{11}+\lambda_{2}Y_{12}+\cdots +\lambda_{8}Y_{18}+S_{1}^{+}=Y_{11},\\ \lambda_{j}\geq 0,j=1,2,\cdots ,n\\ S^{+}\geq 0,S^{-}\geq 0. \end{array}\right. \end{array}$$

According to the judgment theorem of DEA effectiveness, it could be concluded that among the eight departments, 5 departments could achieve DEA effectiveness, namely Department 2, Department 3, Department 4, Department 5, and Department 6. There were three non-DEA effective ones, namely, Department 1, Department 7, and Department 8. Meanwhile, DEA model was used to calculate that ∑_*i*=1_^*θ*^*λi* of all departments were 1, indicating that all departments were in a constant state of return to scale.

Use EXCEL software to solve the weight of each index. Where are the weights of the three input indicators and the two output indicators, respectively. According to the calculation results of the formula, it can be found that only the five management departments of Department 2, Department 3, Department 4, Department 5, and Department 6 have reached the DEA validity. Therefore, the following five departments are sorted according to the formula and department data. Calculate taking Department 2 as an example.

The calculation formula is shown in

(9) $$\begin{array}{c} h^{2t}=\left(\frac{u^{t}Y_{2}}{w^{t}X_{2}},\frac{u^{t}Y_{3}}{w^{t}X_{3}},\frac{u^{t}Y_{4}}{w^{t}X_{4}},\frac{u^{t}Y_{5}}{w^{t}X_{5}},\frac{u^{t}Y_{6}}{w^{t}X_{6}}\right)\text{.} \end{array}$$

**Figure 7:**
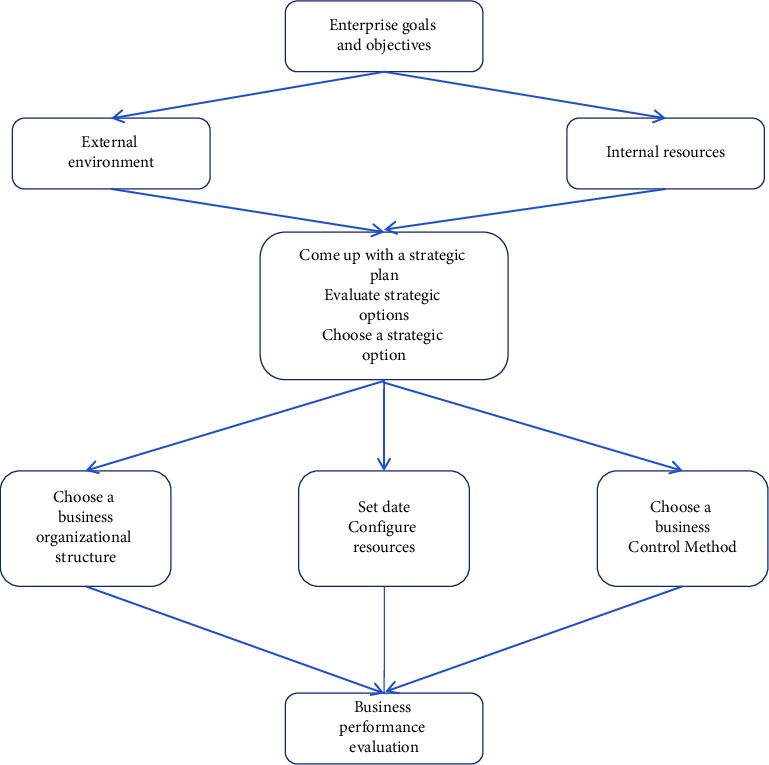
Diagram of performance evaluation relationship.

As the diagonal elements were all 1, it could be seen that all the five departments were DEA effective. However, it could be seen from the results that there were significant differences among these five departments, but the differences could not be seen from the formula. Because different columns were based on different decision-making units. The reason for this inconsistency was that DEA evaluation determined the ranking by determining the weight of the most favorable reference unit. Therefore, it was necessary to synthesize a general sorting conclusion from these different sorting results, which needed to be solved by formula analysis. The total sorting vector H was obtained after the sorting matrix H appeared. The performance evaluation diagram is shown in Figure 7.

For non-DEA effective departments, the adjustment value of the effective frontier to be achieved by the department could be calculated. It could not only reduce the input factors in proportion but also increase the output by strengthening the management. Using the relaxation variable theorem, the target improvement value could be calculated. Starting from Department 1, in order to achieve DEA effectiveness, the output value should be increased by appropriately reducing the corresponding input value. Only in this way could the performance management quality of Department 1 be guaranteed.

There are similarities in the performance evaluation needs of different public administration departments, and the two indicators of the number of complaints and the satisfaction of the masses are the most in need of adjustment. By combining with reality, we need to adjust the salaries of public administration department staff and improve the service level, improve customer service satisfaction, the quality of DEA performance evaluation could be significantly improved. In the actual survey, it was found that taking Department 1 as an example, many users generally reported that there were too few service windows opened by public administration departments for a long time, so they had to queue for more than 30 minutes to handle business most of the time. Slow service speed was contrary to the positioning of serving the people. Long waiting time would not only affect the mood of users but also led to the loss of a large number of users. In addition, in the evaluation of whether the waiters were friendly or not, Department 1 had a low score. In most cases, staff did not take the initiative to help users solve various problems. Moreover, due to the relatively small number of staff, users were often unable to find staff who could help them in the first time when they had demands.

Based on the above analysis, it was suggested that Department 1 strengthen the training of staff service awareness, especially the training of active service awareness. Then the specific work was assigned to each employee and more service windows were opened around the clock to improve the speed of business processing. At the same time, the head of the department should also strengthen the psychology of the staff, so that the staff identify this service from the psychological perspective and a variety of services were provided to users from the heart. When necessary, the incentive system and service supervision mechanism could even be perfected, so as to improve the subjective initiative of staff during service.

The five departments that have achieved DEA effectiveness all have excellent operating results, and among them, the operating results of Department 4 are the best. Among the various business indicators of Department 4, the mass satisfaction index has the greatest contribution to the business results. The actual survey results also confirm the evaluation results of the DEA method. The user satisfaction of Department 4 is so high. According to field observations, it is found that the first impression of Department 4 is that the windows are bright and clean, the environment is relatively comfortable, and the service personnel have a friendly attitude. The smile when treating users can make people feel that they are from the same time, the service speed of the department is very fast, which reduces the waiting time of users to the greatest extent, and because of the clean and comfortable environment, it also creates a psychological hint of high service quality to the masses. Based on the above reasons, it is understandable that the user satisfaction of Department 4 is the highest.

In the input and output index system designed for the social security department, according to the results of empirical analysis, it is found that the two most important indexes are user satisfaction and staff salary. From the data point of view, in order to improve the performance level of the social security department, it is necessary to conduct in-depth research on how to greatly improve user satisfaction and to improve the work system of the public management department with user services as the core. According to modern democratic theory, the political and economic resources necessary for the operation of the government originate from the public. The “user intervention” mechanism is introduced into the performance evaluation and the performance indicators based on it should be determined from the citizens' positions and value choices so that the evaluation results have greater credibility.

## 4. Conclusions

To sum up, this paper analyzes the performance evaluation of public administration departments based on the improved DEA algorithm. By incorporating the DEA model into the analysis, it analyzes the methods for public administration departments to improve performance. Public administrations should devote more energy to customer service as a way to contribute to departmental performance. From the point of view of DEA, public satisfaction and total service cost will affect the performance quality to a certain extent. Only by balancing various influencing factors can the performance evaluation quality of public administration departments be maximized and avoid the impact of performance problems. Long-term development of public administration. It should be noted that although the DEA algorithm is extremely valuable in the performance evaluation of public administration departments, there is still room for optimization to become better.

## Figures and Tables

**Table 1:** The proportion of literature types in performance evaluation of public administration departments.

Research direction	Amount	Percentage	Research direction	Amount	Percentage
Local performance evaluation	206	18.10	Performance evaluation instances	17	1.50%
Performance evaluation and finance	11	0.97	Performance evaluation innovation	20	1.80%
Performance evaluation system	57	5.03	Performance evaluation in different countries	31	2.76%
Performance evaluation value	35	3.09	Performance evaluation methods	60	5.29%
Performance evaluation index system	26	2.29	Performance evaluation system	37	3.21
Other	525	46.30	Total	1134	

**Table 2:** Statistical table of data of social security departments.

Index	Number of services and products (individual)	Unit cost per service (yuan/unit)	Number of services complained of (individual)	Public service satisfaction	Department efficiency
Department 1	233	2135.9	43	4.526	100
Department 2	213	1953.2	41	4.267	100
Department 3	255	1212.4	36	4.311	86.15
Department 4	224	1199.1	37	4.198	87.12
Department 5	198	2315.1	42	4.456	95.11
Department 6	211	1415.2	34	4.512	91.10
Department 7	167	1536.4	37	4.258	88.15
Department 8	189	1752.3	40	4.369	89.12

## Data Availability

The data and code used to support the findings of this study are available from the corresponding author upon request.
